# Revisiting Influenza Vaccination Exemption

**DOI:** 10.3201/eid2410.180304

**Published:** 2018-10

**Authors:** Margaret Ryan, Laurie Duran, Rachel Lee, Sengkham Wu

**Affiliations:** University of California San Diego School of Medicine, San Diego, California, USA (M. Ryan);; Defense Health Agency Immunization Healthcare Branch, San Diego (M. Ryan, L. Duran);; Naval Medical Center, San Diego (M. Ryan, L. Duran, R. Lee, S. Wu)

**Keywords:** immunization, influenza vaccines, vaccination, influenza, viruses, bacteria, exemption

## Abstract

Serious adverse events after immunizations are rare. We review the case of a man who, 50 years earlier, experienced a serious adverse neurologic event 2 weeks after receiving influenza vaccine. He had received no subsequent seasonal influenza vaccinations, but after the risks and benefits were considered, he was vaccinated without adverse event that season.

Neurologic adverse events following immunization (AEFIs), such as encephalitis or acute disseminated encephalomyelitis (ADEM), developing after influenza vaccination have been observed but are rare. It is challenging to determine the causal relationship between an influenza vaccination and an AEFI. A 2011 review by the Institute of Medicine found epidemiologic evidence to be insufficient and mechanistic evidence to be weak for establishing a causal association between influenza vaccination and encephalitis or ADEM (*1*). A more recent review of serious AEFIs found that 4 cases of ADEM had possible causal association with vaccination for the 2009 pandemic influenza A(H1N1) virus (*2*). 

We examined 1 example of an AEFI in a patient who was subsequently issued a medical exemption from future vaccinations. The patient’s original AEFI was documented in 1969 (*3*). Meningoencephalitis developed in the patient, a 29-year-old member of the US military, ≈2 weeks after receiving seasonal influenza vaccine. After a brief hospitalization and supportive care, he recovered without sequelae. The patient was given a medical exemption from subsequent influenza vaccinations for the remainder of his time in the military. For the next 48 years, he declined nearly all vaccinations. (In 2011, the patient did receive 1 dose of a vaccine unrelated to influenza.) 

In September 2017, at 77 years of age, the patient expressed concern to his primary care physician about his level of protection against infections because he was considering moving to an assisted living facility. After discussing risks and benefits with his healthcare providers, he agreed to receive pneumococcal conjugate vaccine 13 in October 2017, followed ≈1 month later by seasonal influenza vaccine (ccIIV4; Flucelvax; Seqirus, Summit, NJ, USA). He reported feeling well over the subsequent 3 months of follow-up and anticipates that in the fall of 2018 he will receive pneumococcal polysaccharide vaccine 23 and seasonal influenza vaccine. 

The influenza vaccine that this patient received in 1969 was a bivalent product that included A2/Aichi/2/68 and B/Massachusetts/3/66 antigens cultured in embryonated chicken eggs (*4*). It is unclear how the 1969 vaccine compares with modern-era influenza vaccines in terms of rates of rare AEFIs and how medical experts assessed causality after the AEFI that resulted in the patient’s exemption from all future influenza vaccinations, nearly 50 years ago. However, AEFI causality assessments have become more rigorous over time, under United States and World Health Organization guidelines (*5**,**6*).

When deciding whether to continue vaccinating a patient who has experienced a serious neurologic AEFI, all available information should be considered, including the licensing of the vaccine and, in the United States, the Centers for Disease Control and Prevention/Advisory Committee on Immunization Practices’ General Best Practice Guidelines for Immunization (*7*). The risk for new or recurrent neurologic events after subsequent vaccination is unknown. 

More cases of encephalitis and ADEM are associated with virus infection than with vaccination. However, recurrence of such events is rare, even after repeated virus infections. Because of this rarity, when relapse of ADEM occurs in adults, it is more likely to be diagnosed as multiple sclerosis than as an independent recurrence of ADEM (*8*). 

Providers may be challenged to determine if, when, and how to administer vaccines to a patient who has had a serious AEFI. Although it may seem easiest and safest to permanently exempt persons from further vaccination, doing so may inappropriately deprive them of disease protection because factors relevant to risk and benefit change over time (*9*). We propose that vaccine exemptions should be revisited regularly, regardless of how long they have been in effect. 
